# Sera selected from national STI surveillance system shows *Chlamydia trachomatis* PgP3 antibody correlates with time since infection and number of previous infections

**DOI:** 10.1371/journal.pone.0208652

**Published:** 2018-12-17

**Authors:** Paula B. Blomquist, Stephanie J. Mighelsen, Gillian Wills, Eleanor McClure, Anthony E. Ades, Daphne Kounali, J. Kevin Dunbar, Myra O. McClure, Kate Soldan, Sarah C. Woodhall, Patrick Horner

**Affiliations:** 1 Blood Safety, Hepatitis, Sexually Transmitted Infections (STI) and HIV Division, Public Health England, London, United Kingdom; 2 Health Protection Research Unit in Blood Borne and Sexually Transmitted Infections at University College London in partnership with Public Health England and in collaboration with London School of Hygiene & Tropical Medicine, London, United Kingdom; 3 Health Protection Research Unit in Evaluation of Interventions at University of Bristol in partnership with Public Health England, Bristol, United Kingdom; 4 Jefferiss Research Trust Laboratories, Section of Infectious Diseases, Wright-Fleming Institute, Faculty of Medicine, Imperial College London, London, United Kingdom; 5 Population Health Science Institute, University of Bristol, Bristol, United Kingdom; University of Texas Health Science Center at San Antonio, UNITED STATES

## Abstract

**Background:**

Seroprevalence surveys of *Chlamydia trachomatis* (CT) antibodies are promising for estimating age-specific CT cumulative incidence, however accurate estimates require improved understanding of antibody response to CT infection.

**Methods:**

We used GUMCAD, England’s national sexually transmitted infection (STI) surveillance system, to select sera taken from female STI clinic attendees on the day of or after a chlamydia diagnosis. Serum specimens were collected from laboratories and tested anonymously on an indirect and a double-antigen ELISA, both of which are based on the CT-specific Pgp3 antigen. We used cross-sectional and longitudinal descriptive analyses to explore the relationship between seropositivity and a) cumulative number of chlamydia diagnoses and b) time since most recent chlamydia diagnosis.

**Results:**

919 samples were obtained from visits when chlamydia was diagnosed and 812 during subsequent follow-up visits. Pgp3 seropositivity using the indirect ELISA increased from 57.1% (95% confidence interval: 53.2–60.7) on the day of a first-recorded chlamydia diagnosis to 89.6% (95%CI: 79.3–95.0) on the day of a third or higher documented diagnosis. With the double-antigen ELISA, the increase was from 61.1% (95%CI: 53.2–60.7) to 97.0% (95%CI: 88.5–99.3). Seropositivity decreased with time since CT diagnosis on only the indirect assay, to 49.3% (95%CI: 40.9–57.7) two or more years after a first diagnosis and 51.9% (95%CI: 33.2–70.0) after a repeat diagnosis.

**Conclusion:**

Seropositivity increased with cumulative number of infections, and decreased over time after diagnosis on the indirect ELISA, but not on the double-antigen ELISA. This is the first study to demonstrate the combined impact of number of chlamydia diagnoses, time since diagnosis, and specific ELISA on Pgp3 seropositivity. Our findings are being used to inform models estimating age-specific chlamydia incidence over time using serial population-representative serum sample collections, to enable accurate public health monitoring of chlamydia.

## Introduction

Genital infection with *Chlamydia trachomatis* (CT) is the most commonly diagnosed bacterial sexually transmitted infection (STI) in England, with more than 200,000 cases of chlamydia reported nationally in 2017 [1]. Most infections are asymptomatic, however an estimated 17% (95% credible interval 6%-29%) of untreated chlamydia in women will result in pelvic inflammatory disease, which can result in significant long term morbidity [2].

England’s National Chlamydia Screening Programme (NCSP) has been implemented in all regions of England since 2008. It aims to reduce chlamydia transmission and the consequences of untreated infection through opportunistic testing and treatment of 15–24 year olds in clinical and non-clinical settings [3]. Evaluation of the screening programme has proven challenging. Routine reporting of chlamydia testing and diagnoses allows monitoring of numbers and positivity trends but not incidence and prevalence of infection, because the population tested is typically at higher risk of STIs, more symptomatic, and more engaged with health providers than the general population [4, 5], and is also variable over time and place. Two large population-based studies of CT prevalence have been completed in the UK [6, 7], but these are too costly to repeat often, challenging to execute, and cannot detect modest changes in prevalence due to the sample size available and the room for variation in participation biases.

Seroepidemiology offers an alternative evaluation method. The presence of anti-CT serum antibodies is an indicator of both recently acquired and previous infection, which can inform the estimation of cumulative incidence [8–10]. We have developed two sensitive and specific enzyme-linked immunosorbent assays (ELISA) based on the *C*. *trachomatis*-specific antigen Pgp3, which is transcribed from the highly conserved CT plasmid and has been found to be highly immunogenic. Pgp3 has also not been found in human *C*. *pneumonia* isolates and antibody to Pgp3 does not cross react with *C*. *pneumonia* proteins [10, 11]. The indirect ELISA has a sensitivity of 73.8% (95% CI 66.5–79.9) to detect a previously diagnosed infection, and specificity of 97.6% (95% CI: 96.2 to 98.6%) [11]. The double-antigen ELISA was subsequently developed and allows for the detection of lower antibody levels. It was found to have a higher sensitivity of 82.9% (95% CI 77.0–88.8%) and specificity of 97.8% (95% CI 96.5–99.1%) [10] when tested against the same standards as the indirect ELISA.

Effective CT control would be expected to lead to a reduction in incidence. Pgp3 seroprevalence, as a marker of age-specific cumulative incidence, has been explored as a means of evaluating the NCSP in two studies in England; the first using anonymously tested residual serum from health services [5, 12] and the second using population-representative specimens collected through a national household survey [8]. Both studies noted possible decreases in age-specific seroprevalence since the implementation of the NCSP [5, 8]. However, interpretation of how age-specific seroprevalence relates to cumulative incidence is complicated by evidence that antibody detection is affected by the time interval since diagnosis and treatment, the number of prior CT infections, and the ELISA used [9–11]. Further work quantifying the relationship between natural history and antibody response will enable improved interpretation of serological data [5, 8–10].

England’s health data landscape provides an opportunity to conveniently conduct a serological study. All genitourinary medicine (GUM) clinics in England submit detailed clinical data to the GUMCAD STI Surveillance System, the national dataset on sexual health services and STI diagnoses in England [13]. The data contain pseudo-anonymised identifiers that can be linked to residual sera, i.e. sera leftover after diagnostic blood testing, resulting in clinically characterised serological specimens. The potential of two Pgp3-specific ELISAs as epidemiological tools to evaluate chlamydia screening was investigated using these resources. Specifically, selected residual sera were collected, anonymised and linked to GUMCAD data, and tested for Pgp3-specific IgG antibody in order to examine the relationship between chlamydia Pgp3 seropositivity and a) the number of known previous chlamydia diagnoses, and b) the time since a known diagnosis.

## Method

This research was undertaken as part of the National Institute of Health Research (NIHR) Health Protection Research Unit (HPRU) in Blood Borne and Sexually Transmitted Infections at University College London and the HPRU in Evaluation of Interventions at University of Bristol.

### Source of data and serum

As a matter of routine practice, GUM clinic attendees are tested for infection with CT, *Neisseria gonorrhoeae*, syphilis and HIV. While chlamydia and gonorrhoea are tested using a nucleic acid amplification test (NAAT) applied to urine specimens or genital swabs, HIV and syphilis testing require a blood specimen to be collected. Leftover blood, i.e. residual serum, is commonly stored by the diagnostic laboratories following testing, and may be available for other purposes such as research. Testing information and results are stored in GUMCAD by Public Health England (PHE).

In this study, GUMCAD was used to select study sites and serum specimens. Six sites were recruited to participate, each consisting of a GUM clinic and their affiliated diagnostic laboratory. Site selection was pragmatic, based on each clinic having a large number of patients diagnosed with chlamydia each year, and/or >70% of clinic attendees diagnosed with chlamydia having a blood test on the same day. The selected laboratories also routinely stored residual sera for up to two years, thus increasing the likelihood of specimens being available.

### Selection of serum specimens

We selected serum specimens from women aged 16 to 44 years old with at least one positive chlamydia NAAT test (hereafter referred to as a chlamydia diagnosis) in their GUMCAD records. Serum specimens were identified by record of an HIV or syphilis blood test (which indicated that the associated residual serum should be in storage). To ensure serum specimen availability, patients must have visited any of the participating GUM clinics within the two years prior to specimen selection (29/5/2013 to 30/9/2015).

**Fig 1:**
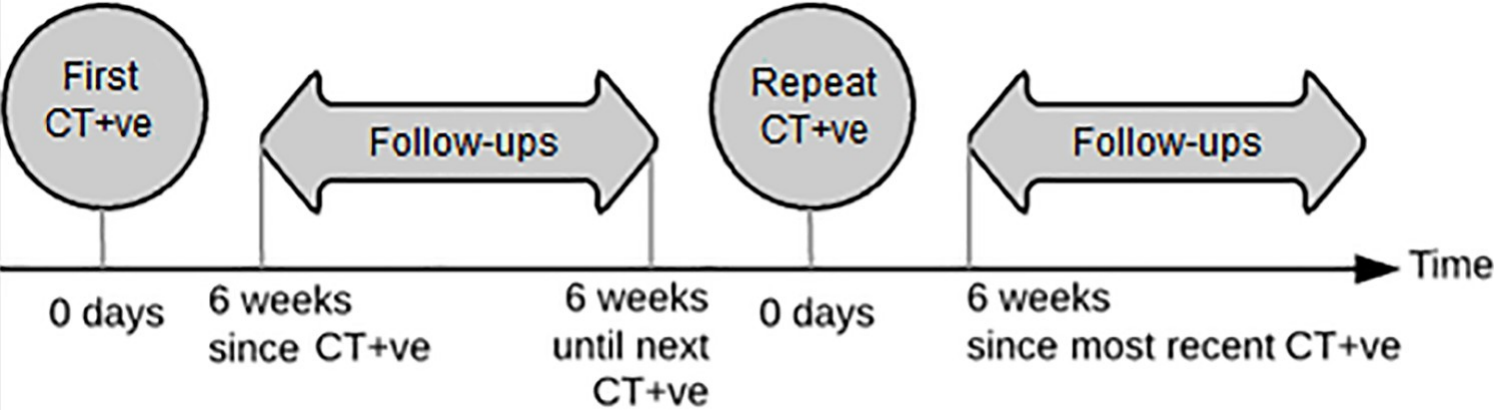
Schematic demonstrating collected specimen types.

Two groups of specimens were selected: diagnosis (“CT+ve” for ‘chlamydia positive’) specimens and follow-up specimens. CT+ve specimens were those taken from patients on the same day as a chlamydia diagnosis; either their first recorded diagnosis (“first CT+ve”) or a repeat diagnosis (“repeat CT+ve”). Follow-up specimens were those taken from a patient on the same day as a negative chlamydia NAAT test (CT-ve) and at least six weeks after a prior chlamydia diagnosis (Fig 1). This minimum six week gap between patients’ positive and negative NAAT tests is a feature of the GUMCAD dataset to ensure the tests are for different episodes of care.

We requested a maximum of 200 residual specimens from each laboratory for each of three quarterly rounds of collection, in line with the number of specimens considered feasible by each lab. To ensure a variety of specimen types within this limit, we prioritised repeat CT+ves and follow-ups after a repeat CT diagnosis. We also prioritised specimens from the same patients to enable a longitudinal analysis.

### Specimen collection

The lists of selected specimens were sent to recruited laboratories, who retrieved and couriered the serum specimens (minimum 90μL) on ice to PHE, where they were stored at 4°C for a maximum of two weeks before processing. Specimens were further pseudo-anonymised with a bespoke algorithm that created new identifiers (IDs) based on the original IDs. This ensured specimens could not be linked back to the patient at the time of testing, however could be grouped by patient for longitudinal analysis. Specimens were stored at -20°C then were sent on dry ice to Imperial College London for testing.

### Serological testing

All specimens were tested using an indirect ELISA and a double-antigen ELISA, as previously described [10, 11]. Thresholds for seropositivity were set at 0.473 OD_450_ [11] for the indirect ELISA and at 0.44 OD_450_ for the double-antigen ELISA [10].

### Statistical analysis

Two cross-sectional analyses were carried out:

1. Seropositivity by diagnosis number: Seropositivity was calculated among specimens taken on the same day as a patient’s first, second, or third (or more) recorded CT diagnosis.
2. Seropositivity by time since CT diagnosis: Seropositivity was calculated among follow-up specimens taken at different time points since the (most recent) recorded CT diagnosis.

A nested longitudinal analysis was also undertaken. Seroconversion and seroreversion were investigated using pairs of first CT+ve and follow-up specimens from the same women, where available.

Seroconversion was defined as testing seronegative with the first CT+ve specimen and then seropositive with a follow-up specimen; seroreversion was defined as being seropositive with the first CT+ve specimen but seronegative with a follow-up specimen. We calculated the proportion of women who seroconverted and seroreverted, and generated Kaplan-Meier plots to investigate rates of seroconversion and seroreversion within one year after a chlamydia diagnosis using both ELISAs.

All statistical analyses were carried out using STATA version 13 (StataCorp. College Station, TX).

### Ethics

Ethical approval was obtained from the Greater Manchester West Research Ethics Committee (reference 15/NW/0042). Patient consent was not required as no additional specimens were taken outside of routine patient management, and specimens and data were non-identifiable to the researchers.

## Results

### Specimen retrieval

There were 130,597 clinic attendances by 16 to 44 year old women within the dates of specimen availability (29/5/2013 to 30/9/2015) at the six study clinics. 50,927 of these attendances included both a CT test and a blood test for either HIV or syphilis, indicating availability of a residual serum specimen with an associated CT NAAT result. When looking across each patient’s clinical history, we identified 6,782 eligible specimens, taken on the same day as or after a CT diagnosis. We requested 2,151 serum specimens and 1,731 specimens (80%) were received and tested on both ELISAs. Of these, 919 specimens were CT+ve and 812 were follow-ups (see S1 Fig in Supplementary material for specimen collection flow chart). This included matched sets of 154 first CT+ve and 189 follow-up specimens from 154 women, for the nested longitudinal analysis.

Follow-up specimens and repeat CT+ve specimens were from slightly younger patients compared to first CT+ve specimens (Table 1).

**Table 1:** Number of serum specimens, number of contributing patients, and age of patients by specimen type.

Specimen type	Number of collected serum specimens	Median age of patient providing specimen (Range)

**CT+ve**	**919**	**21 (16–44)**
First CT+ve	673	21 (16–44)
Repeat CT+ve	246	21 (16–39)
Second CT+ve	179	21 (16–38)
Third or more CT+ve	67	21 (18–39)
**Follow-up**	**812**	**21 (16–35)**
After a first diagnosis	646	20 (16–33)
After a repeat diagnosis	166	21 (16–35)
**Total**	**1,731**	**20 (16–44)**

### Seropositivity by number of cumulative CT diagnoses

**Fig 2:**
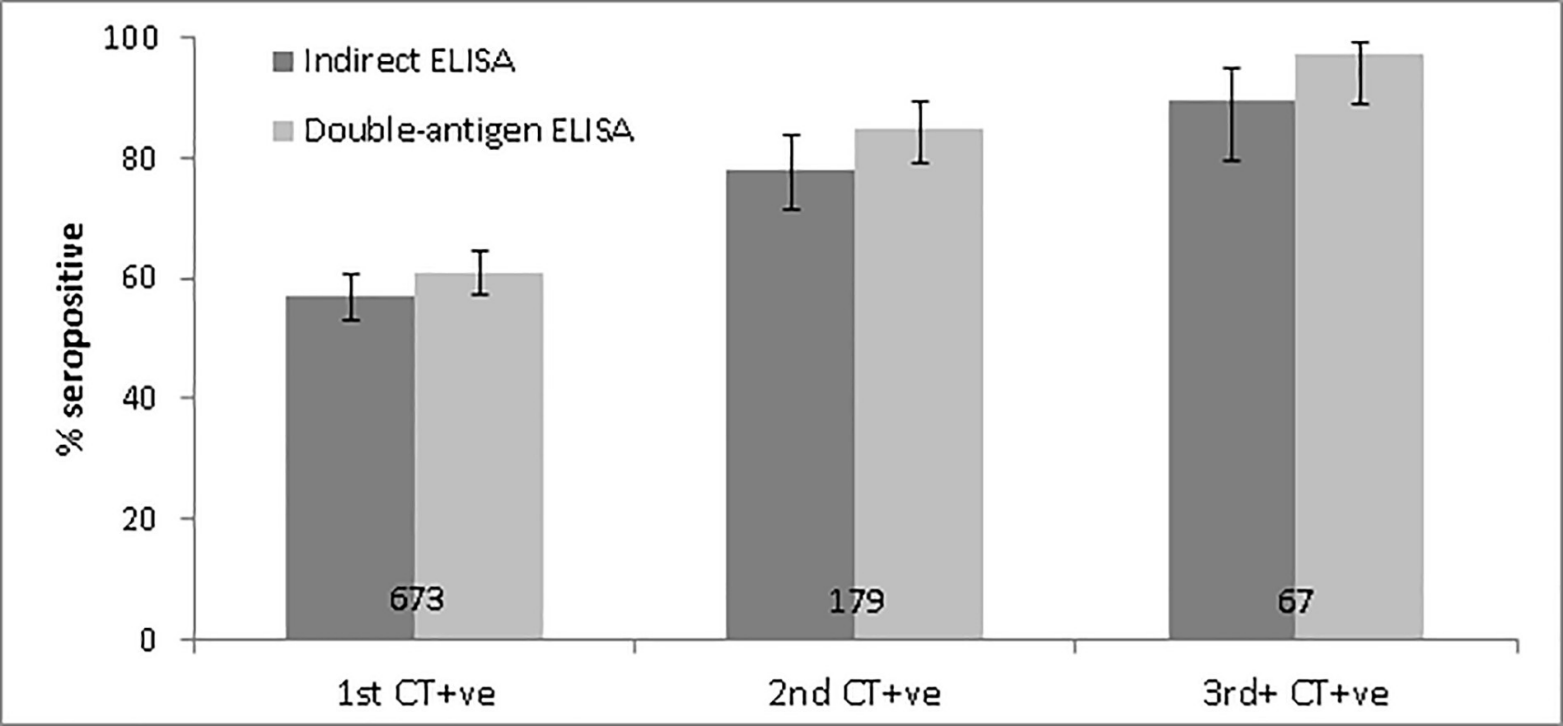
Pgp3 seropositivity on the indirect and double-antigen ELISAs among specimens taken on the same day as a patient’s first, second, and at least third chlamydia diagnosis (Denominator labelled on bar. Error bars represent 95% confidence intervals).

Among first CT+ve specimens, seropositivity was 57.1% (95% CI: 53.2–60.7) with the indirect ELISA and 61.1% (95% CI: 57.3–64.7) with the double-antigen ELISA. Seropositivity increased with total cumulative number of diagnoses using both ELISAs, and reached 89.6% (95% CI: 79.3–95.0) and 97.0% (95% CI: 88.5–99.3) seropositivity with the indirect and double-antigen ELISAs among specimens taken on the day of a third or more chlamydia diagnosis (Fig 2; see S1 Table in Supplementary material for full results). Seropositivity was consistently lower with the indirect ELISA compared to the double-antigen ELISA, although confidence intervals overlap.

### Seropositivity by time since CT diagnosis in first/repeat infections

**Fig 3:**
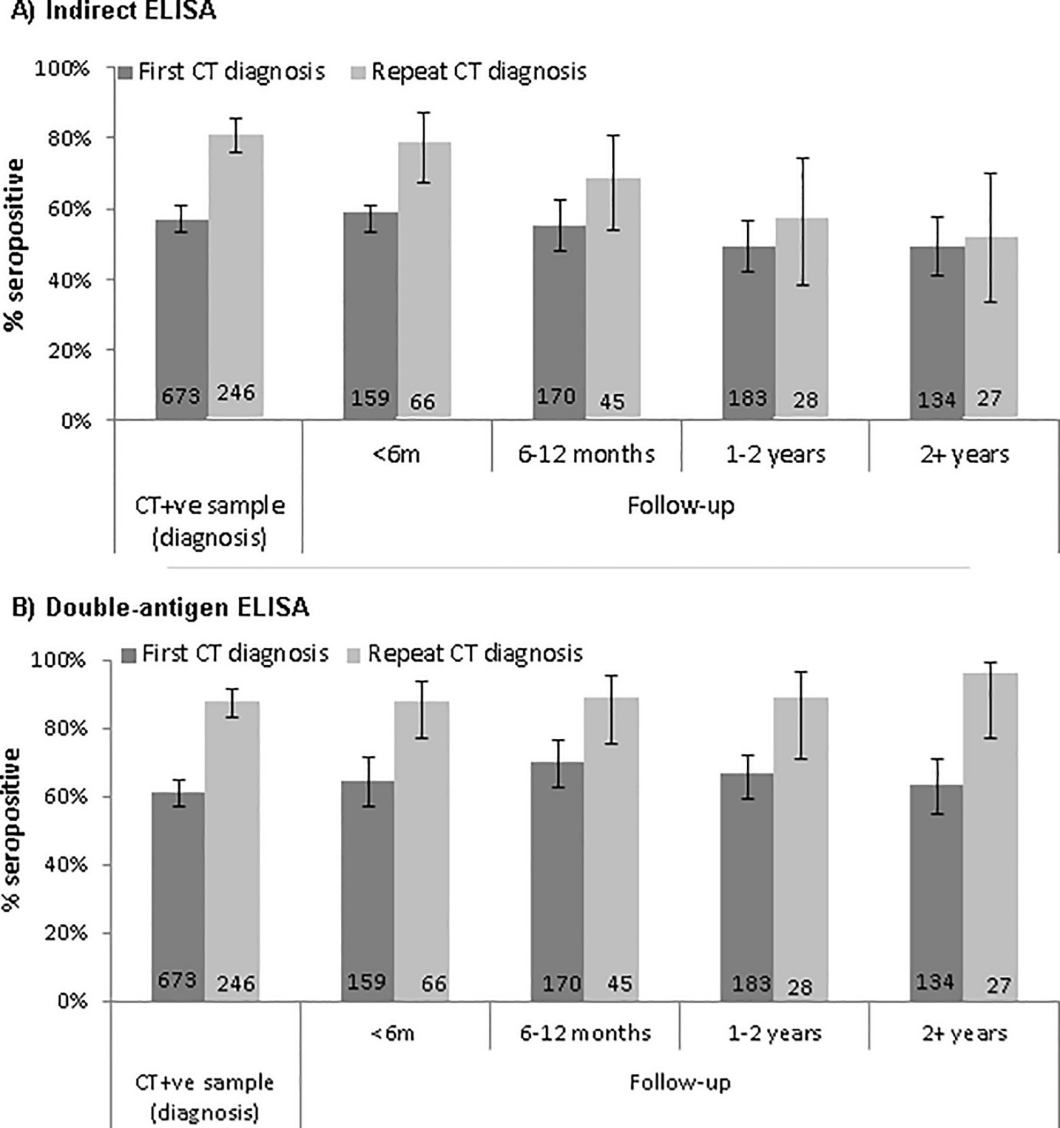
**Pgp3 seropositivity by time since most recent chlamydia diagnosis on a) the indirect ELISA and b) the double-antigen ELISA** (Denominator labelled on bar. Error bars represent 95% confidence intervals).

In our cross-sectional analysis, seropositivity measured using the indirect ELISA decreased with time since a CT diagnosis (Fig 3A). The decrease was particularly evident in specimens from patients with a repeat diagnosis: seropositivity was 81.3% (95% CI: 75.9–85.7) in repeat CT+ve specimens, but decreased to 51.9% (95% CI: 33.2–70.0) in follow-up specimens taken two or more years after a repeat CT diagnosis. In contrast, there was no significant change in seropositivity over time since diagnosis when measured using the double-antigen ELISA (Fig 3B; S1 Table).

### Seroconversion and seroreversion in the longitudinal specimens

In the nested longitudinal analysis, paired first CT+ve and follow-up specimens were available from 154 women. Among these women, 27 had two or more follow-up specimens available.

**Fig 4:**
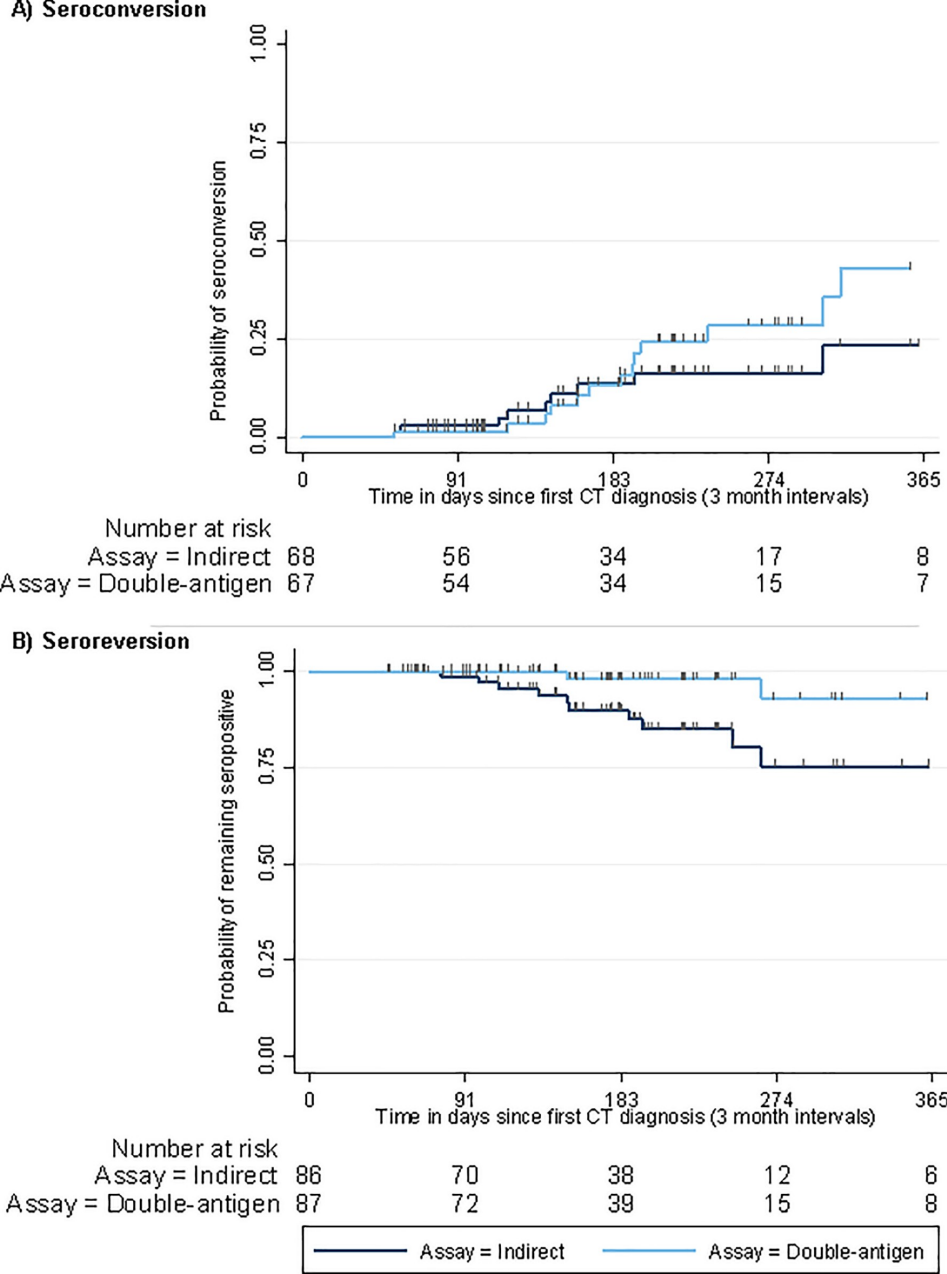
**Kaplan-Meier curves on time to first a) seroconversion and b) seroreversion at a follow-up attendance in nested longitudinal study** (y axis limited to 365 days; small vertical tick marks show censoring times).

Among patients whose first CT+ve specimens tested negative for Pgp3 antibody on the indirect and double-antigen ELISAs, 14.7% (95% CI: 8.0–25.6) and 22.4% (95% CI: 13.8–34.2) respectively had seroconverted in a subsequent follow-up (not necessarily the first follow-up) (Table 2; see S2 Table in supplementary material for full results). Rates of seroconversion detected were similar with both ELISAs within the first six months of diagnosis but diverged at around 6 months, with more individuals seroconverting with the double-antigen ELISA compared to the indirect ELISA (Fig 4A).

**Table 2:** Percentage of patients who seroconverted or seroreverted at follow-up in nested longitudinal analysis, by ELISA type.

	Pgp3 seronegative CT+ve specimen			Pgp3 seropositive CT+ve specimen		
	No. women	No. women seroconverted	% (95% CI)	No. women	No. women seroreverted	% (95% CI)
Indirect	68	10	14.7 (8.0–25.6)	86	10	11.6 (6.3–20.5)
Double-antigen	67	15	22.4 (13.8–34.2)	87	3	3.4 (1.1–10.4)

Among patients whose first CT+ve specimen tested positive for Pgp3 antibody on the indirect and double-antigen ELISAs,11.6% (95% CI: 6.3–20.5) and 3.4% (95% CI: 1.1–10.4) respectively had seroreverted in a subsequent follow-up specimen (not necessarily the first follow-up), indicating that the level of Pgp3 antibodies were below the limits of detection of the ELISA. Rates of seroreversion detected using the indirect and double-antigen ELISAs diverged at around 3 months after a chlamydia diagnosis (Fig 4B). However, these plots should be interpreted with caution as the numbers at-risk decrease notably over time.

## Discussion

Using residual sera collected from GUM clinic attendees, we found that Pgp3 seropositivity increased with cumulative number of chlamydia diagnoses using both indirect and double-antigen ELISAs, and decreased over time after diagnosis when tested on the indirect ELISA but not on the double-antigen ELISA. Seropositivity was consistently higher on the double-antigen ELISA, particularly in repeat CT+ve specimens and follow-up specimens six months or more after a diagnosis. Seroconversion was more often detected with the double-antigen compared to the indirect ELISA, and seroreversion occurred earlier on the indirect ELISA, indicating that the indirect ELISA was less successful at detecting the lower level of antibodies present after a presumed treatment.

This was a large study investigating how Pgp3 seropositivity in women varies according to the combined effect of number of known previous infections, time since diagnosis, and assay type. It also explored whether observations on how seropositivity changes since time from diagnosis are similar in a large cross-sectional study compared to a longitudinal cohort study. Efficient use of surveillance data and stored residual sera allowed relatively rapid collection of specimens with detailed characterisation of specimens in terms of the patients’ history of chlamydia tests and diagnoses. The use of data derived from clinic records—as opposed to patient questionnaires—avoided inaccuracy due to selection bias or recall bias of previous CT infection.

Although GUMCAD is an invaluable resource, there are limitations to its use. GUMCAD only provides the clinical history of a patient within a particular clinic and our longitudinal study relied on individuals re-attending; due to the anonymous nature of these clinical attendances, there was no possibility of linking data between clinics or actively following patients up. Consequently, individuals’ undiagnosed chlamydia infections or diagnoses carried out in other clinics are unknown. We also do not have access to information about and cannot control for behavioural factors such as number of sexual partners, self-reported history of chlamydia in another setting, or age of sexual debut, previously identified to be risk factors associated with chlamydia [7]. We were able to use specimens from 154 women as part of the longitudinal analysis, however the true date of seroconversion or seroreversion is unknown, as it would have occurred at some point between the diagnosis and follow-up attendance. Finally, GUM clinic attendees also differ from the general population in terms of demographic makeup and clinical history: they are understood to have more incident infections which are more likely to be symptomatic [2, 5].

Our observations of Pgp3 seropositivity in women diagnosed with chlamydia are comparable to other studies. In a national health survey of the general population of England, Woodhall et al observed that 65.5% of women aged 16–44 who reported a prior chlamydia diagnosis were Pgp3 seropositive using the double-antigen ELISA [8]. This is comparable to the 71.2% seropositivity with the double-antigen assay among all follow-ups observed in this study. Our finding of sustained seropositivity for up to two years with the double-antigen ELISA is also consistent with a retrospective cohort study in New Zealand, which showed high seropositivity with the double-antigen ELISA for at least 12 years following a chlamydia diagnosis [10]. Seroconversion and seroreversion were also noted in a small number of women over time, as seen in this study.

Some studies found slightly higher seropositivity, however this is likely related to differences in the study populations. In a study of GUM-attending women with a chlamydia diagnosis at least one month prior, Pgp3 seropositivity was 73.8% using the indirect ELISA [11]. A later study investigating the specimens in more detail observed a higher seropositivity of 82.9% using the double-antigen ELISA [10]. This is higher than the respective 56.3% and 71.2% seropositivity among all follow-up specimens we observed with the indirect and double-antigen ELISAs. An additional study, again using the same specimens, observed a sustained high seropositivity with the indirect ELISA after a repeat diagnosis [9], whereas our study suggested that seropositivity decreased in this group of specimens. The specimens used in these studies were from older women compared to this study, and it likely that a higher proportion of them had upper genital tract disease and/or repeat chlamydia infections. As observed here and in other studies [14, 15], these factors are associated with higher antibody titre and together can explain why we observed lower seropositivity.

A further possible reason for the difference is that women in our study were diagnosed earlier in the course of infection. Researchers who found higher seropositivity reported patient waiting times of up to two weeks [9], whereas walk-in chlamydia testing was common in our recruited clinics. Early testing may have led to more false positive diagnoses, due to the detection of genetic material from non-viable *C*. *trachomatis* that did not establish a true infection [2]. This has previously been referred to as passive infection [2], and would result in underestimated Pgp3 seropositivity due to an inflated denominator of CT+ve specimens. Additionally, it has been suggested that early testing and treatment could result in an arrested immune response with a reduction in the production of antibodies against CT [16]. This effect is likely to be small, if any, given that Pgp3 seropositivity has now repeatedly been shown to increase with the number of previous infections, suggesting that the immune system is indeed primed after an initial infection, even if treated. However, as the true timing of chlamydia exposure is unknown in this study, it is not possible to comment on whether these factors did affect Pgp3 seropositivity in this study. These suggestions highlight the complexities of interpreting serological findings.

These results demonstrate, as previously reported [9–11], that the Pgp3 antibody is a sensitive marker of previous infection but that detection performance differs by assay methodology. It may be appropriate to use the indirect ELISA to screen samples in larger scale studies and then use the double-antigen to retest samples with low absorbance values that are more difficult to quantify (between 0.1–1.0 OD_450_), as it requires a 25-fold larger volume of serum. Methods of analysis considering the effects of single and repeat infections as well as time since infection should be developed. These considerations should also be applied when assessing performance of other assays, to ensure comparison between CT antibody assays. The similarity of the cross-sectional and nested longitudinal results also suggests that the more convenient cross-sectional methodology may be sufficiently accurate to capture serological responses over time.

This work furthers the evidence-base for using chlamydia serology for public health evaluation. This paper details a methodology for serum-based studies using residual serum and national surveillance data, and a chlamydia serum bank is being established using the remaining characterised specimens for use by other researchers. The collection methodology could be extended to further understand the relationship between antibody response and CT infection and related complications and could also be applied to other STIs. Future use of enhanced GUMCAD data, with additional sexual behaviour data, would allow more detailed clinical and behavioural characteristics to be included with collected specimens. These data are now being incorporated into a multiparameter evidence synthesis modelling at the University of Bristol, to estimate age-specific incidence and cumulative incidence using serial population-based sera in England. Findings from this modelling work will be incorporated into the evaluation of the NCSP and used to inform and optimise chlamydia control in England.

## Supporting information

S1 Fig Flow chart of specimen retrieval. (TIF)

S1 File Codebook for the minimal study dataset. (DOCX)

S2 File Minimal study dataset–CSV file. (CSV)

S3 File Minimal study dataset–DTA file. (DTA)

S1 Table Seropositivity on indirect and double-antigen assay, by number of cumulative CT diagnoses and time since most recent CT diagnosis. (DOCX)

S2 Table Nested longitudinal study results among women with CT+ve and at least one follow-up specimen, presented by ELISA and time since CT+ve: (A) Seroconversions among women with seronegative CT+ve specimen (B) Seroreversions among women with seropositive CT+ve specimen.
(DOCX)
