# Correction: Sera selected from national STI surveillance system shows *Chlamydia trachomatis* PgP3 antibody correlates with time since infection and number of previous infections

**DOI:** 10.1371/journal.pone.0216193

**Published:** 2019-04-24

**Authors:** Paula B. Blomquist, Stephanie J. Migchelsen, Gillian Wills, Eleanor McClure, Anthony E. Ades, Daphne Kounali, J. Kevin Dunbar, Myra O. McClure, Kate Soldan, Sarah C. Woodhall, Patrick Horner

The second author’s name is spelled incorrectly. The correct name is: Stephanie J. Migchelsen. The correct citation is: Blomquist PB, Migchelsen SJ, Wills G, McClure E, Ades AE, Kounali D, et al. (2018) Sera selected from national STI surveillance system shows *Chlamydia trachomatis* PgP3 antibody correlates with time since infection and number of previous infections. PLoS ONE 13(12): e0208652. https://doi.org/10.1371/journal.pone.0208652
