# Supplementary figures and images for: Sera selected from national STI surveillance system shows Chlamydia trachomatis PgP3 antibody correlates with time since infection and number of previous infections
Source: PLoS One. 2018 Dec 17;13(12):e0208652. doi: 10.1371/journal.pone.0208652 (PMC6296657; doi:10.1371/journal.pone.0208652)

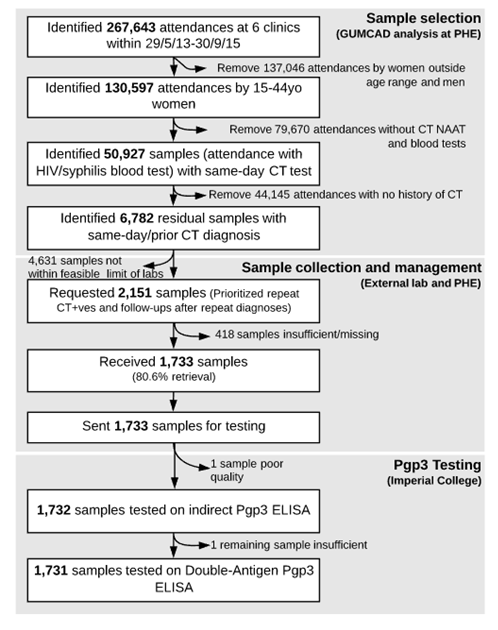

Supplement: S1 Fig — (TIF) [file pone.0208652.s001.tif]
