# Supplementary material for: Sera selected from national STI surveillance system shows Chlamydia trachomatis PgP3 antibody correlates with time since infection and number of previous infections
Source: PLoS One. 2018 Dec 17;13(12):e0208652. doi: 10.1371/journal.pone.0208652 (PMC6296657; doi:10.1371/journal.pone.0208652)
Supplement: S1 File — (DOCX) [file pone.0208652.s002.docx]

| **NIHR chlamydia Pgp3 study – codebook**  This codebook should be used when analysing the dataset ‘*CT serology dataset – sharing’* (available as *dta* or *csv* file. Note that the *csv* file presents the categorical variables in string format, with the labels for values).  This dataset has been fully anonymised and cannot be linked back to the original GUM data. Its publication has been given ethics and local Caldicott approval.  Please contact [paula.blomquist@phe.gov.uk](mailto:paula.blomquist@phe.gov.uk) for queries | | | |
| --- | --- | --- | --- |
| Variable description | Variable name | Variable values and labels | Notes |
| Unique patient identifier | ID | <integer> | (anonymised and unlinked from original GUM records) |
| Age group | age_group | 1 – 16-19  2 – 20-24  3 – 25-29  4 – 30-44 |  |
| Chlamydia NAAT test result | ct_result | 0 – Negative  1 – Positive |  |
| Sample type | ct_specimen | 1 – First CT+ve  2 – Repeat CT+ve  3 – Follow- up sample |  |
| Cumulative number of CT+ve tests | ct_cumulativepos | <integer> |  |
| Cumulative number of CT+ve tests (3 categories) | ct_cumulativepos_cat | 1 – One  2 – Two  3 – Three or more |  |
| Cumulative number of CT+ve tests among follow-up samples (2 categories) | ct_cumulativepos_FU | 1 – One previous CT+ve  2 – Two or more previous CT+ve |  |
| Days since most recent CT+ve test | ct_time | <integer> | 0 days if on the same day as a CT+ve NAAT test. Time is measured according to most recent CT+ve test in GUM records, regardless of specimen availability. |
| Indirect Pgp3 ELISA result | ELISA_indirect | 0 – Negative  1 – Positive |  |
| Double-antigen Pgp3 ELISA result | ELISA_da | 0 – Negative  1 – Positive |  |
