# Supplementary material for: Sera selected from national STI surveillance system shows Chlamydia trachomatis PgP3 antibody correlates with time since infection and number of previous infections
Source: PLoS One. 2018 Dec 17;13(12):e0208652. doi: 10.1371/journal.pone.0208652 (PMC6296657; doi:10.1371/journal.pone.0208652)
Supplement: S1 Table — (DOCX) [file pone.0208652.s005.docx]

S1 Table. Seropositivity on indirect and double-antigen assay, by number of cumulative CT diagnoses and time since most recent CT diagnosis

|  | **N** | **Seropositive on indirect ELISA** | | | | | | | | | **Seropositive on double-antigen ELISA** | | | | | | | | | | | | | | |
| --- | --- | --- | --- | --- | --- | --- | --- | --- | --- | --- | --- | --- | --- | --- | --- | --- | --- | --- | --- | --- | --- | --- | --- | --- | --- |
|  |  | **n** | **%** | **95% Confidence interval** | | | | | | | **n** | | | | **%** | **95% Confidence interval** | | | | | | | | | |
| CT+ve specimens | | | |  |  |  | |  | |  | |  |  | | | |  | |  | |  | |  | |  |
| **Total CT+ve** | 919 | 584 | 63.6 | (60.4 | | - | 66.6) | | | | 628 | | | 68.3 | | (65.2 | | | | - | | 71.3) | | | |
| **First CT+ve** | 673 | 384 | 57.1 | (53.3 | | - | 60.8) | | | | 411 | | | 61.1 | | (57.3 | | | | - | | 64.7) | | | |
| **Repeat CT+ve** | 246 | 200 | 81.3 | (75.9 | | - | 85.7) | | | | 217 | | | 88.2 | | (83.5 | | | | - | | 91.7) | | | |
| 2^nd^ CT+ve | 179 | 140 | 78.2 | (71.5 | | - | 83.7) | | | | 152 | | | 84.9 | | (78.9 | | | | - | | 89.5) | | | |
| 3^rd^+ CT+ve | 67 | 60 | 89.6 | (79.6 | | - | 95.0) | | | | 65 | | | 97.0 | | (88.7 | | | | - | | 99.3) | | | |
| Follow-up specimens | | | | | | | | |  | |  | | |  | |  | |  | |  | |  | |  | |
| By time since CT diagnosis | | | | | | | | |  | |  | | |  | |  | |  | |  | |  | |  | |
| Total | 812 | 457 | 56.3 | (52.8 | | - | 59.7) | | | | 578 | | | 71.2 | | (68.0 | | | | - | | 74.2) | | | |
| <6m | 225 | 146 | 64.9 | (58.4 | | - | 70.9) | | | | 161 | | | 71.6 | | (65.3 | | | | - | | 77.1) | | | |
| 6-12 months | 215 | 125 | 58.1 | (51.4 | | - | 64.6) | | | | 159 | | | 74.0 | | (67.6 | | | | - | | 79.4) | | | |
| 1-2 years | 211 | 106 | 50.2 | (43.4 | | - | 57.0) | | | | 147 | | | 69.7 | | (63.1 | | | | - | | 75.6) | | | |
| 2+ years | 161 | 80 | 49.7 | (42.0 | | - | 57.4) | | | | 111 | | | 68.9 | | (61.3 | | | | - | | 75.7) | | | |
| By time after **first** CT diagnosis | | | | | | | | |  | |  | | |  | |  | |  | |  | |  | |  | |
| Total | 646 | 344 | 53.2 | (49.4 | | - | 57.1) | | | | 429 | | | 66.4 | | (62.7 | | | | - | | 70.0) | | | |
| <6m | 159 | 94 | 59.1 | (53.3 | | - | 60.8) | | | | 103 | | | 64.8 | | (57.0 | | | | - | | 71.8) | | | |
| 6-12 months | 170 | 94 | 55.3 | (47.7 | | - | 62.6) | | | | 119 | | | 70.0 | | (62.7 | | | | - | | 76.4) | | | |
| 1-2 years | 183 | 90 | 49.2 | (42.0 | | - | 56.4) | | | | 122 | | | 66.7 | | (59.5 | | | | - | | 72.1) | | | |
| 2+ years | 134 | 66 | 49.3 | (40.9 | | - | 57.7) | | | | 85 | | | 63.4 | | (54.9 | | | | - | | 71.2) | | | |
| By time after **repeat** CT diagnosis | | | | | | | | |  | |  | | |  | |  | |  | |  | |  | |  | |
| Total | 166 | 113 | 68.1 | (60.5 | | - | 74.8) | | | | 149 | | | 89.7 | | (84.1 | | | | - | | 93.6) | | | |
| <6m | 66 | 52 | 78.8 | (67.2 | | - | 87.1) | | | | 58 | | | 87.9 | | (77.5 | | | | - | | 93.9) | | | |
| 6-12 months | 45 | 31 | 68.9 | (53.9 | | - | 80.8) | | | | 40 | | | 88.9 | | (75.7 | | | | - | | 95.4) | | | |
| 1-2 years | 28 | 16 | 57.1 | (38.3 | | - | 74.1) | | | | 25 | | | 89.3 | | (71.0 | | | | - | | 96.6) | | | |
| 2+ years | 27 | 14 | 51.9 | (33.2 | | - | 70.0) | | | | 26 | | | 96.3 | | (77.2 | | | | - | | 99.5) | | | |
