# Supplementary material for: Sera selected from national STI surveillance system shows Chlamydia trachomatis PgP3 antibody correlates with time since infection and number of previous infections
Source: PLoS One. 2018 Dec 17;13(12):e0208652. doi: 10.1371/journal.pone.0208652 (PMC6296657; doi:10.1371/journal.pone.0208652)
Supplement: S2 Table — Nested longitudinal study results among women with CT+ve and at least one follow-up specimen, presented by ELISA and time since CT+ve: (A) Seroconversions among women with seronegative CT+ve specimen (B) Seroreversions among women with seropositive CT+ve specimen. (DOCX) [file pone.0208652.s006.docx]

S2A Table. Nested longitudinal study results: Seroconversions among women with seronegative CT+ve specimen and at least one follow-up specimen, presented by ELISA and time since CT+ve

| Time since date of seronegative CT+ve specimen | **Serology results of specimens (cross-sectional analysis)** | | | **Seroconversions among women (cohort analysis)** | | | | |
| --- | --- | --- | --- | --- | --- | --- | --- | --- |
|  | No. follow-ups within time period | No. seropositive follow-ups within time period | % seropositive within time period | No. women with follow-up sample in time period | No. women who seroconverted in time period | Cumulative no. women with follow-up samples by end of time period | Cumulative no. women who seroconverted by end of time period | Cumulative % of women who seroconverted by end of time period |
| **Indirect ELISA** | | | | | | | | |
| < 6 months | 44 | 7 | 15.9% | 41 | 7 | 41 | 7 | 17.1% |
| 6m - 1 year | 30 | 3 | 10.0% | 22 | 2 | 63 | 9 | 14.3% |
| 1 - 2 years | 9 | 1 | 11.1% | 5 | 1 | 68 | 10 | 14.7% |
| Total | 83 | 11 | 13.3% | 68 | 10 | 68 | 10 | 14.7% |
| **Double-antigen ELISA** | | | | | | | | |
| < 6 months | 44 | 6 | 13.6% | 41 | 5 | 41 | 5 | 12.2% |
| 6m - 1 year | 32 | 7 | 21.9% | 22 | 7 | 63 | 12 | 19.0% |
| 1 - 2 years | 8 | 2 | 25.0% | 4 | 3 | 67 | 15 | 22.4% |
| Total | 84 | 15 | 17.9% | 67 | 15 | 67 | 15 | 22.4% |

S2B Table. Nested longitudinal study results: Seroreversions among women with seropositive CT+ve specimen and at least one follow-up specimen, presented by ELISA and time since CT+ve

| Time since date of seropositive CT+ve specimen | **Serology results of specimens (cross-sectional analysis)** | | | **Seroreversions among women (cohort analysis)** | | | | |
| --- | --- | --- | --- | --- | --- | --- | --- | --- |
|  | No. follow-ups within time period | No. seronegative follow-ups within time period | % seronegative within time period | No. women with follow-up sample in time period | No. women who seroreverted in time period | Cumulative no. women with follow-up samples by end of time period | Cumulative no. women who seroreverted by end of time period | Cumulative % of women who seroreverted by end of time period |
| **Indirect ELISA** | | | | | | | | |
| < 6 months | 61 | 6 | 9.8% | 60 | 6 | 60 | 6 | 10.0% |
| 6m - 1 year | 37 | 5 | 13.5% | 23 | 4 | 83 | 10 | 12.0% |
| 1 - 2 years | 8 | 2 | 25.0% | 3 | 0 | 86 | 10 | 11.6% |
| Total | 106 | 13 | 12.3% | 86 | 10 | 86 | 10 | 11.6% |
| **Double-antigen ELISA** | | | | | | | | |
| < 6 months | 61 | 1 | 1.6% | 60 | 1 | 60 | 1 | 1.7% |
| 6m - 1 year | 35 | 1 | 2.9% | 23 | 1 | 83 | 2 | 2.4% |
| 1 - 2 years | 9 | 2 | 22.2% | 4 | 1 | 87 | 3 | 3.4% |
| Total | 105 | 4 | 3.8% | 87 | 3 | 87 | 3 | 3.4% |
